# A prospective study to assess the clinical utility of serum HER2 extracellular domain in breast cancer with HER2 overexpression

**DOI:** 10.1007/s10549-016-4000-z

**Published:** 2016-10-05

**Authors:** Nathalie Reix, Charlotte Malina, Marie-Pierre Chenard, Jean-Pierre Bellocq, Stéphanie Delpous, Sébastien Molière, Anthony Sevrin, Karl Neuberger, Catherine Tomasetto, Carole Mathelin

**Affiliations:** 1 ICube UMR 7357, Université de Strasbourg/CNRS, Fédération de Médecine Translationnelle de Strasbourg (FMTS), Strasbourg, France; 2 Laboratoire de Biochimie et Biologie Moléculaire, Hôpitaux Universitaires de Strasbourg, 1 place de l’Hôpital, 67091 Strasbourg, France; 3 Unité de Sénologie, Hôpitaux Universitaires de Strasbourg, 1 avenue Molière, 67098 Strasbourg, France; 4 Service de Pathologie, Hôpitaux Universitaires de Strasbourg, 1 avenue Molière, 67098 Strasbourg, France; 5 Department of Functional Genomics and Cancer, Institut de Génétique et de Biologie Moléculaire et Cellulaire, CNRS UMR 7104, INSERM U964, Université de Strasbourg, Illkirch, France; 6 Department of Imaging, Hôpitaux Universitaires de Strasbourg, 1 avenue Molière, 67098 Strasbourg, France; 7 Quantmetry, 55 rue La Boetie, 75008 Paris, France

**Keywords:** Breast cancer, HER2 positive, Extracellular domain ECD, Prognosis factor, Monitoring, Trastuzumab

## Abstract

**Purpose:**

We explored the clinical utility of human epidermal growth factor receptor-2 extracellular domain (HER2/ECD) in patients treated for an invasive breast cancer with HER2 overexpression.

**Methods:**

We prospectively studied HER2/ECD levels in the sera of 334 women included between 2007 and 2014, all treated with trastuzumab. HER2/ECD levels were measured at diagnosis, during treatments, and along the follow-up. We investigated the relationship of HER2/ECD with other clinicopathological parameters at diagnosis, its prognosis value, and its utility during the monitoring of a neoadjuvant treatment and the follow-up.

**Results:**

Elevated HER2/ECD at diagnosis correlated positively with parameters associated with tumor aggressiveness. Disease-free survival of non-metastatic patients was significantly shorter in patients with high HER2/ECD at diagnosis (HR = 13.6, 95 % CI 1.6–113.6, *P* < 0.0001). Progression-free survival of metastatic patients was better for patients with low HER2/ECD (HR = 2.6, 95 % CI 1.2–5.3, *P* = 0.033). A multivariate analysis revealed that HER2/ECD level at diagnosis was an independent prognosis factor. During neoadjuvant therapy, a significant decrease in HER2/ECD was reported only for the complete histological response group (*P* = 0.031). During the follow-up, HER2/ECD helped predict relapse, disease progression, and metastases before imaging in 18.6 % cases of the studied cohort.

**Conclusions:**

HER2/ECD is a prognosis factor that is valuable in evaluating the neoadjuvant treatment efficiency. HER2/ECD also appears to be a helpful surveillance biomarker for the early diagnosis of relapses and to predict the fate of metastases. This study brings evidences to support the use of HER2/ECD in the management of HER2-positive breast cancer.

## Introduction

About 15–20 % breast carcinomas have an overexpression of human epidermal growth factor receptor-2 (HER2) [1]. This overexpression has been associated with a more aggressive disease, a poor clinical prognosis and the therapeutic success of trastuzumab [2]. HER2 has an extra-cellular domain (ECD) mainly produced by HER2 cleavage and released into circulation [3]. Several observations suggest that this cleavage is of clinical importance [4]. Indeed, the truncated form is 10 to 100-fold more oncogenic than the full-length HER2 form [5]. A wide range of studies over the last 20 years reports that high HER2/ECD levels detected in the serum are associated with tumor aggressiveness, a less positive prognosis, and disease progression [6, 7]. Consequently, elevated HER2/ECD levels may represent a subgroup of HER2-positive tumors with a higher level of HER2 cleavage that is associated to a more aggressive clinical course. Therefore, HER2/ECD could be a biomarker that helps identify this subgroup of tumor and improve the risk stratification.

However, the results on the clinical importance of HER2/ECD are based on studies that have not all been conducted according to the international recommendations for tumor markers [8] and that did not respect every specific recommendation existing for HER2/ECD quantification. Indeed, according to the Food and Drug Administration, HER2/ECD must be quantified with one of the two validated immunoenzymatic methods; the ELISA manual method and the automatic method Immuno-1, both developed by Siemens Healthcare Diagnostics. These assays use the same set of two monoclonal antibodies, the same calibration material and a unique pathological threshold ≥15 ng/mL [9, 10]. Furthermore, pathologies that are associated with an elevation of HER2/ECD in 40–60 % of patients [11] must be excluded, specifically hepatic ones. A review of the literature shows that there are few prospective studies meeting these criteria [12] and that more studies are needed to consolidate the utility of this assay in the management of breast cancer.

On the basis of these findings, we conducted a prospective study in accordance with the international guidelines and used an FDA-approved assay. In this study, we analyzed HER2/ECD circulating levels in a series of patients treated for an invasive breast cancer with HER2 overexpression, and examined their association with pathological parameters, prognosis, therapeutic response, and disease progression.

## Patients and methods

The study was conducted in accordance with the REMARK criteria [8].

### Study design

**Fig. 1:**
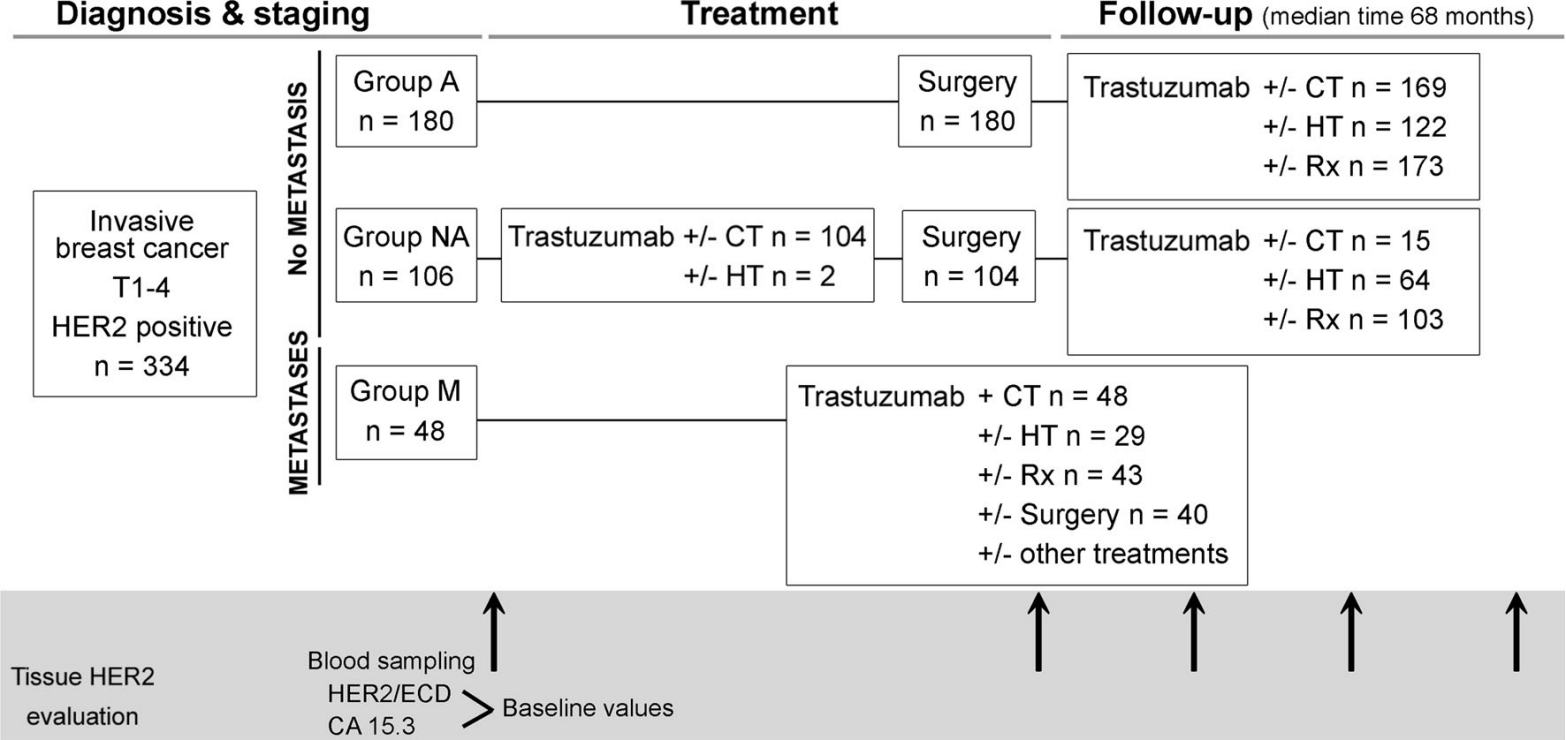
Design of the study. All enrolled patients had an invasive breast cancer (IBC) with HER2 overexpression. They were divided into three groups: Group adjuvant (*A*), Group neoadjuvant (*NA*) and Group metastases (*M*)

This clinical study was prospective. The cohort consisted of 334 consecutive Caucasian women having a positive HER2 breast cancer treated with trastuzumab. Patients were enrolled from January 2007 to April 2014 at a single French regional hospital: the Hôpitaux Universitaires de Strasbourg. All patients gave their written informed consent. The French ethic committee gave its agreement for the conduction of this study. Exclusion criteria were negative HER2 tumor and hepatic diseases. The study design is presented in Fig. 1 and explained below.

### Clinical and anatomopathological parameters

Clinical parameters obtained for all patients included lymph node status, metastatic status, age and menopausal status at diagnosis. The primary tumor characteristics included size and histological type. Histological grade was evaluated using the Elston–Ellis modification of the SBR grading system [13]. Lymph node involvement was analyzed using haematoxylin-eosin (HE) and immunohistochemistry (IHC) (anti-cytokeratin) in cases where the sentinel lymph node procedure was used. Estrogen receptors (ER) and progesterone receptors (PR) were examined using IHC with the monoclonal antibodies SP1 for ER (Ventana Confirm anti-ER SP1) and 1E2 (Ventana Confirm anti-ER SP1anti-PR 1E2) for PR. These were quantified using the international H-score value [14]. Vascular invasion (VI) was defined as the presence of cancer cell microemboli in lymphatic and/or blood vessels histologically estimated around primary tumors [15].

### Tissue HER2 status determination

Tissue HER2 status was analyzed by immunohistochemistry (IHC) (clone Ventana pathway HER2 4B5) in all tumors and by in situ hybridization (ISH) for score 2+ tumors by IHC (INFORM HER2 dual ISH DNA probe cocktail, Ventana) according to the ASCO and to experts recommendations [1, 16, 17]. The laboratory regularly contributes to the National Breast Pathology External Quality Assessment scheme.

### HER2/ECD and CA 15.3 analyses

From January 2007 to October 2008, serum samples were analyzed for HER2/ECD using a manual ELISA assay kit (Siemens Healthcare Diagnostics). The assay has a limit of quantification (LOQ) of 1.5 ng/mL, a linear measuring range up to 35 ng/mL, and day-to-day variability at 4.1, 10.2, and 20.8 ng/mL of 9.1, 8.6, and 6.7 %, respectively. It was replaced starting on 7 October 2008 by an automated sandwich immunoassay using direct chemiluminescent technology (ADVIA Centaur HER2/neu assay, Siemens Healthcare Diagnostics) with LOQ of 0.5 ng/mL, a linear measuring range up to 350 ng/mL and an interassay CV of 5.7 % at 13.9 ng/mL and 4.3 % at 97.6 ng/mL. Serum HER2/ECD was considered positive when ≥15 ng/mL for both the assays. A strong correlation between the results of automated and the manual assays was described [18].

Measurements of carbohydrate antigen 15.3 (CA 15.3) were performed using an automated immunoassay on Kryptor (Brahms, Thermo scientific). CA 15.3 serum values above 30 U/mL were considered abnormal.

### HER2/ECD and CA 15.3 during the monitoring of metastatic breast cancer

During the follow-up of the 48 patients of the metastatic group, we studied the correlation between HER2/ECD and CA 15.3 level changes and the evolution of target lesions (bone, liver, lung, brain metastases) on CT, PET, or scintigraphy. Change in HER2/ECD level was evaluated during a three-month period before imaging. Each imaging evaluation with associated HER2/ECD was considered. In consequence, if one patient had undergone several imaging examinations with associated HER2/ECD measurements during the follow-up, then each biological-imaging correlation was analyzed independently.

We considered that HER2/ECD level was consistent with metastasis progression when the increase between the result obtained 3 months before imaging and the result obtained just before imaging was at least 10 % or when the level remained elevated. Conversely, we considered that HER2/ECD was correlated with metastasis regression when the decrease was at least −10 % or when the level remained negative. The same analysis was applied to CA 15.3. These values (±10 %) were based on the day-to-day variability results as described in the previous paragraph.

### Statistical analyses

To assess the association among clinicopathological variables and the levels of HER2/ECD, *P* values were calculated using the χ^2^ test.

ANOVA I with a Student–Newman–Keuls post hoc test was used after logarithm transformation of data to evaluate HER2/ECD level differences between groups.

Univariate investigations of the relationship between baseline HER2/ECD levels and overall survival (OS), disease-free survival (DFS) for non-metastatic patients and progression-free survival (PFS) for metastatic patients were conducted using the Kaplan–Meier method. The log-rank test was used to test the differences between survival curves. Hazard ratios (HR) with confidence intervals (95 % CI) were computed through a multivariate analysis using Cox proportional hazards regressions, adjusting for the following variables: grade, nodal status, vascular invasion, estrogen and progesterone receptors, HER2 status, and CA 15.3.

HER2/ECD level evolution during neoadjuvant therapy and during the monitoring of therapy in the metastatic group was analyzed using the Wilcoxon test for paired samples.

For all tests, a *P* value < 0.05 was considered as significant. Statistical analyses were performed with the MedCalc software version 12.2 (Mariakerke, Belgium), and the R software version 2.7 for computing the Cox regression.

## Results

### Clinical and biological characteristics of breast cancer subsets

Patients and tumor characteristics at the diagnosis are detailed in Table 1. These data show that some of the cohort characteristics such as the mean age at diagnosis, the percentage of node positive patients, the grade and tumor type distributions, and the rate of positive hormone receptors are consistent with the European breast cancer population characteristics [19, 20].

**Table 1:** Characteristics at diagnosis of the 334 patients and 336 breast cancer tumors included in the series

	Adjuvant group	Neo-adjuvant group	Metastatic group	Total
Number	180	106	48	334
Age (years)				
Average (range)	55 (27–98)	53 (24–84)	58 (23–91)	55 (23–98)
Median (SD)	53 (12)	53 (13)	55 (15)	53 (13)
<45 years ≥45 to <55 years	28 69	30 31	8 14	66 (19.8 %) 114 (34.1 %)
≥55 years	83	45	26	154 (46.1 %)
Breast tumor size (mm)				
Average (range)	19.5 (1–60)	42.5 (8–100)	36.8 (11–80)	29.1 (1–100)
Median (SD)	17 (11)	40 (21.4)	30 (22.1)	25 (19.6)
ND	0	2	3	5
Number of patients with positive node	76	33	25	134 (40.1 %)
Average (range)	1.03 (1–3)	1.33 (1–2)	1.6 (1–3)	1.21 (13)
Median (SD)	1 (0.23)	1 (0.48)	1 (0.71)	1 (0.48)
Grade				
Low grade	12	3	1	16 (4.8 %)
Intermediate grade	60	46	16	122 (36.5 %)
High grade	107	55	26	188 (56.3 %)
ND	1	2	5	8
Tumor type				
Ductal	171	101	30	302 (89.9 %)
Lobular	6	4	9	19 (5.7 %)
Ductal and lobular	2	1	0	3 (0.9 %)
Tubular	2	0	0	2 (0.6 %)
Micropapillary	0	0	1	1 (0.3 %)
Medullar	0	0	2	2 (0.6 %)
ND	0	0	7	7 (2.0 %)
Predictive factors				
HR+	123	64	29	216 (64.7 %)
HR−	57	42	19	118 (35.3 %)
ND	1	0	1	2
HER2 3+	156	101	47	304 (91 %)
HER2 2 + ISH+	24	5	1	30 (9 %)
HER2/ECD (ng/mL)				
<15	168	90	25	283 (84.7 %)
≥15	12	16	23	51 (15.3 %)
Average (range)	9.6 (2.4–25.6)	11.5 (5–53.9)	42.3 (6.4–714)	14.9 (2.4–714)
Median (SD)	8.8 (3.1)	9.2 (7.7)	14.7 (105.2)	9.3 (41.4)
CA 15.3 (U/mL)				
<30	167	84	27	278 (83.2 %)
≥30	13	22	21	56 (16.8 %)
Average (range)	18.6 (6.3–77.6)	24.6 (5–107.4)	177.2 (9.3–2542)	43 (5–2542)
Median (SD)	16.6 (8.9)	20.7 (14.2)	26.0 (423.3)	19 (168.6)

*ND* not done, *HR* hormone receptors

Patients were divided into three groups according to the pathological characteristics of breast cancer at diagnosis: (1) patients devoid of distant metastasis first treated with surgery (group Adjuvant (A)), (2) patients devoid of distant metastasis first treated with systemic therapy by trastuzumab and chemotherapy or hormone therapy (group Neo-adjuvant (NA)), (3) patients with distant metastases (group Metastases (M)) (Fig. 1). Among the 48 metastatic patients, 35 cases involved one organ (13 cases of bone metastasis, 9 cases of liver metastasis, 2 cases of brain metastasis, 9 cases of lung metastasis, 2 cases of skin metastasis).

### Elevated HER2/ECD levels at diagnosis were associated with advanced breast cancer

HER2/ECD at diagnosis was examined in order to highlight differences in HER2/ECD levels between patient groups (A, NA or M). The results are the following: in group A, 6.7 % of values were higher than 15 ng/mL, 15.1 % in group NA, and 47.9 % in group M (Table 1). We noticed a significant difference in HER2/ECD levels between the three groups: a higher HER2/ECD level was observed for the group M (*P* < 0.001). Thus, we can conclude that patients with distant metastases presented more elevated HER2/ECD levels at the time of diagnosis compared to non-metastatic patients.

### Among relationships with histological parameters at diagnosis, HER2/ECD was not correlated with HER2 overexpression intensity (HER2 2+ vs HER2 3+)

We observed relationships between HER2/ECD levels at diagnosis alongside the most discriminating prognosis factors in term of patient care. Statistical associations of HER2/ECD with histological and biological parameters are presented in Table 2. The χ^2^ *P* indicated a correlation between high HER2/ECD levels at diagnosis and vascular invasion, metastases, negativity of estrogen receptors, and CA 15.3 level ≥30 U/mL (Table 2). We observed no significant correlation between high HER2/ECD levels and menopausal status, lymph node status, grade, or progesterone receptor status. We also noticed a lack of correlation between HER2/ECD levels at diagnosis and HER2 overexpression intensity. This can be explained by the fact that tumors scored 3+ can be quite heterogeneous with cancer cells expressing high and low level of HER2 that can co-exist in the same tumor, or with cancer cells diluted in a rich tumor stroma. Thus, HER2/ECD is not a substitute of tumor HER2 status determination.

**Table 2:** Correlation between HER2/ECD at diagnosis and age, N, M, VI, grade, ER, PR, CA 15.3, and HER2 status

	HER2/ECD <15	HER2/ECD ≥15	*P* value
Number	283	51	
Age (years)			
<45	56 (85 %)	10 (15 %)	0.0865
≥45 to <55	103 (90 %)	11 (10 %)	
≥55	124 (81 %)	30 (19 %)	
Lymph node status			
N0	162 (86 %)	26 (14 %)	0.9449
N+ (pTNM)	114 (85 %)	20 (15 %)	
No data^a^	7	5	
Vascular invasion			
VI−	239 (83 %)	49 (17 %)	**0.0458**
VI+	44 (96 %)	2 (4 %)	
Metastatic status			
M0	258 (90 %)	28 (10 %)	**<0.0001**
M+	25 (52 %)	23 (48 %)	
Grade			
Low grade	15 (94 %)	1 (6 %)	0.5832
Intermediate grade	103 (84 %)	19 (16 %)	
High grade	158 (84 %)	30 (16 %)	
No data^a^	7	1	
Estrogen receptor status			
ER−	95 (79 %)	25 (21 %)	**0.0283**
ER+	188 (88 %)	26 (12 %)	
Progesterone receptor status			
PR−	182 (84 %)	34 (16 %)	0.8691
PR+	101 (86 %)	17 (14 %)	
Tissue HER2 status			
HER2 2+ ISH+	27 (90 %)	3 (10 %)	0.5946
HER2 3+	256 (84 %)	48 (16 %)	
CA 15.3			
<30	252 (91 %)	26 (9 %)	**<0.0001**
≥30	31 (55 %)	25 (45 %)	

^a^ Patients not operated or no Roche score on breast carcinoma

*N* lymph node, *VI* vascular invasion, *M* metastasis; *ER* estrogen receptor, *PR* progesterone receptor

### HER2/ECD level at diagnosis was an independent prognosis factor for overall survival

**Fig. 2:**
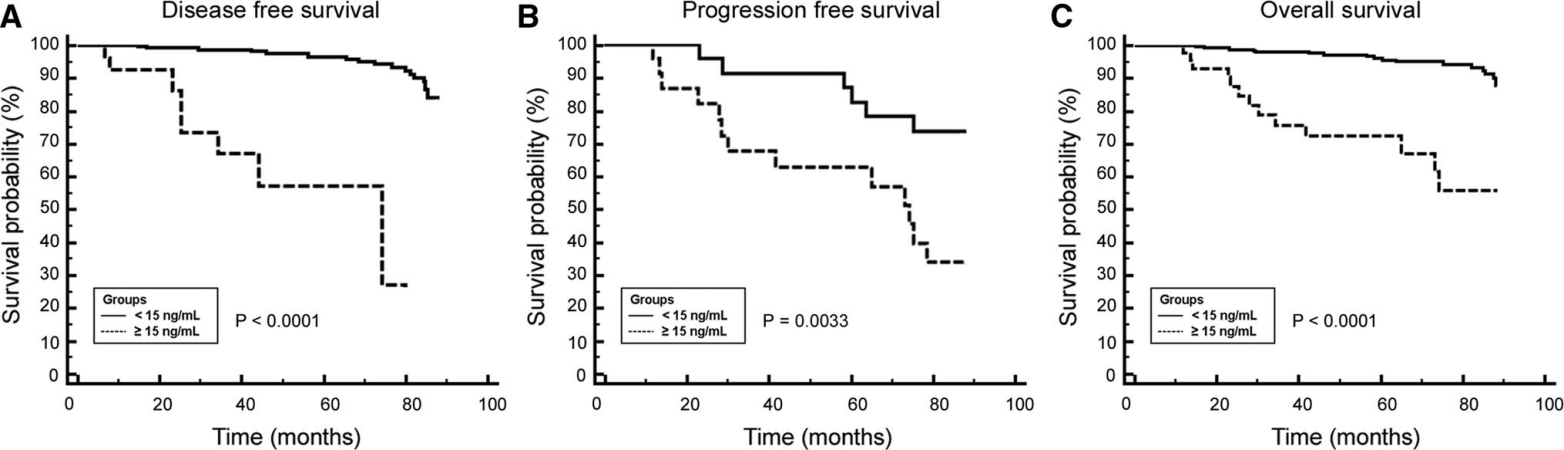
Kaplan–Meier estimates for (**A**) disease-free, (**B**) progression-free, and (**C**) overall survival according to serum HER2/ECD levels at diagnosis

Testing whether HER2/ECD is a prognosis factor or not was done by analyzing the patients survival. The median duration of the follow-up was 68 months for the entire cohort (mean 61 months, range 6–88 months). Among the 163 non-metastatic patients followed at least 5 years after the diagnosis, 97.5 % are still alive. In the 32 patients of the metastatic group followed at least 5 years, the overall survival is 62.5 %. The relationship between HER2/ECD levels at diagnosis and survival is shown in Fig. 2. DFS of non-metastatic patients was significantly shorter in patients with high HER2/ECD levels (≥15 ng/mL) compared to patients with low HER2/ECD levels (<15 ng/mL) (HR = 13.6, 95 % CI 1.6–113.6, *P* < 0.0001) (Fig. 2A). PFS of metastatic patients was better for patients with a low HER2/ECD level (HR = 2.6, 95 % CI 1.2–5.3, *P* = 0.033) (Fig. 2B). OS was significantly shorter for patients with high HER2/ECD levels (HR = 8.90, 95 % CI 2.8–28.7, *P* < 0.0001) (Fig. 2C). Multivariate analyses revealed that HER2/ECD was an independent prognosis factor for OS. Indeed, high serum HER2/ECD levels were strongly associated with worse overall survival (HR = 4.88, 95 % CI 1.6–14.9, *P* = 0.010). In conclusion, high HER2/ECD levels were associated with the overall survival duration for the HER2 overexpressing breast tumors of this study.

### A significant decrease of HER2/ECD level during trastuzumab neaoadjuvant therapy was an indicator of the treatment efficacy

**Fig. 3:**
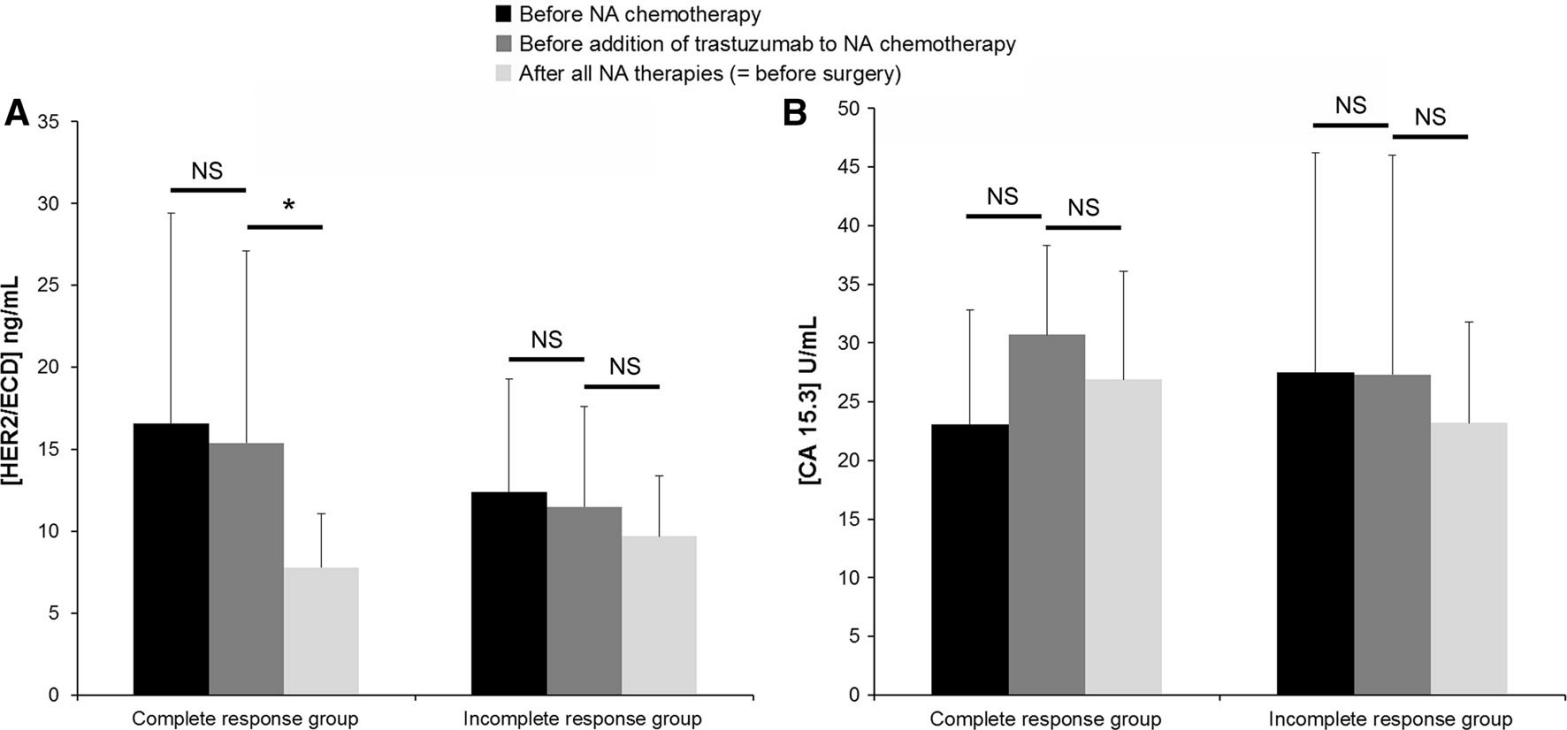
Kinetics of (**A**) HER2/ECD and (**B**) CA 15.3 during neoadjuvant (NA) treatment in the complete (n = 7) and incomplete (n = 16) histological response groups Biomarker levels were examined at three time points during the NA treatment; first, before NA chemotherapy beginning, then, before the addition of trastuzumab to the chemotherapy, and finally, at the end of all NA treatments right before surgery

We followed the evolution of HER2/ECD levels during the neoadjuvant treatment of group NA in order to test whether the changes of this biomarker were correlated with the efficacy of the neoadjuvant therapy. The median duration of a neoadjuvant therapy was 4.3 months (SD 2.0). In the complete histological response group, the results showed no significant evolution of HER2/ECD levels during the NA chemotherapy administered alone (*P* = 0.094). These same results revealed a significant decrease of HER2/ECD levels during the period ranging from the moment trastuzumab was added to NA chemotherapy to the end of the NA treatments (*P* = 0.031, Fig. 3A). In the incomplete histological response group, we found no significant difference in HER2/ECD levels during the neoadjuvant treatment. In each group, we found no difference in CA 15.3 levels (Fig. 3B). These data were obtained from a small effective of patients (<20 in the incomplete and complete response groups). Indeed, the study was designed to test HER2/ECD before and after NA therapy. Consequently, HER2/ECD was not systematically quantified before trastuzumab introduction. Thereby, the question about the utility of HER2/ECD as a useful circulating biomarker to follow the trastuzumab therapy efficacy during the neoadjuvant treatment of invasive breast cancer with HER2 overexpression, while CA 15.3 cannot, remains to be addressed in future studies.

### HER2/ECD was useful to detect certain cancer recurrences at an early stage

We focused on the usefulness of HER2/ECD for an early detection of recurrence. During the follow-up, we observed 70 events (progression, metastases, and death) in the cohort of 334 patients. An increase of HER2/ECD without any change in CA 15.3 levels helped to predict relapse, disease progression, and metastasis before imaging in 18.6 % cases. In 35.7 % of cases, we noticed a concomitant increase of HER2/ECD levels and CA 15.3 before the diagnosis of relapses or metastases. In 8.6 % of cases, CA 15.3 increased before the diagnosis of cancer recurrence without any change in HER2/ECD levels. However, for 37.1 % of patients, neither HER2/ECD nor CA 15.3 helped to predict the occurrence of metastases. To conclude, HER2/ECD provides in some situations an additional information over tumor marker CA 15.3 for the detection of early cancer recurrence.

### HER2/ECD was useful to predict the fate of metastases

**Fig. 4:**
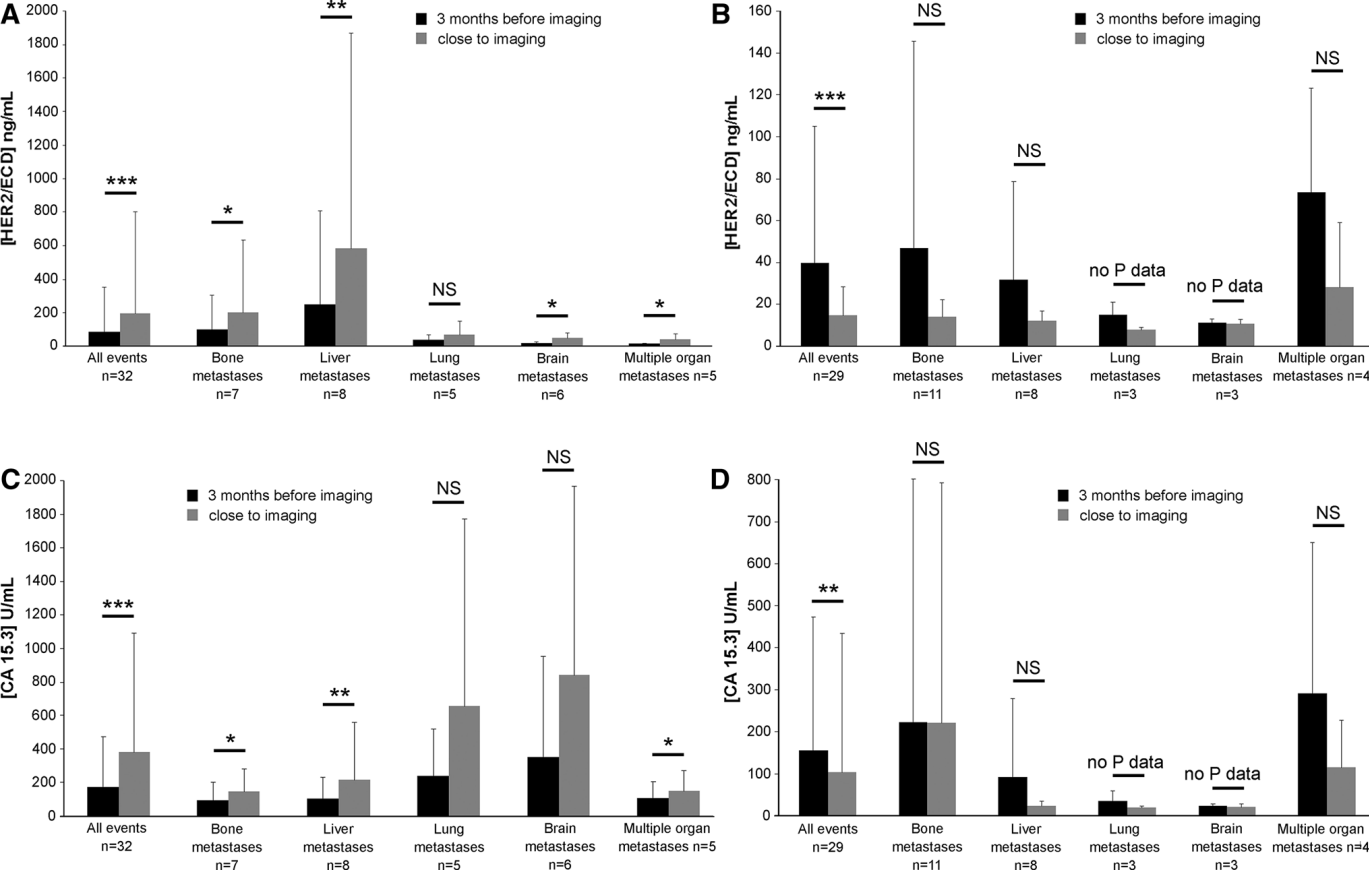
HER2/ECD and CA 15.3 changes during the 3 months before medical imaging of metastasis breast cancer. Correlation of biomarkers with imaging results: HER2/ECD level changes during metastasis (**A**) progression and (**B**) regression. CA 15.3 level changes during metastasis (**C**) progression and (**D**) regression. No P data: due to small sample size

During the follow-up of the 48 patients of the metastatic group, we assessed if changes in HER2/ECD levels (increase or decrease) 3 months before imaging were consistent with imaging results (metastasis progression or regression). Sixty-five imaging results were obtained. Figure 4A and B show that during metastasis progression (Fig. 4A) or regression (Fig. 4B), HER2/ECD was significantly modified (levels increased or decreased, respectively) considering all events. We observed the same significant results for CA 15.3 evolution (Fig. 4C, D). When focusing on specific metastasis localizations, HER2/ECD was significantly increased before imaging conclusion of metastasis progression for bone, liver, brain, and multiple organ metastases but not for lung metastasis progression (Fig. 4A). Figure 4B and D revealed non-significant decreases of HER2/ECD or CA 15.3 for each specific metastasis localization. However, to considerer therapeutic efficacy, we observed not only biomarker changes but also if the level remained elevated or below the reference value. Thus, each HER2/ECD evolution was consistent with imaging conclusion in 94 % against 85 % for CA 15.3 (Table 3). HER2/ECD predicted the metastasis evolution in 100 % for liver and multiple organ metastases (Table 3). A lower performance to predict bone, lung, and brain metastasis progression or regression was observed (90, 89, and 90 % of correlation, respectively) (Table 3). It appeared that CA 15.3 had lower percentages of correlation (Table 3) but discrepancies in both HER2/ECD and CA 15.3 were never observed in a single event, suggesting once again the complementarity of these biomarkers. In consequence, HER2/ECD was useful to predict the fate of metastases.

**Table 3:** Observation in metastasis breast cancer of the concordance between HER2/ECD and CA 15.3 changes and subsequent medical imaging conclusion (progression or regression of metastases) (an event = a medical imaging)

	All events *n* = 65		Bone metastases *n* = 20		Liver metastases *n* = 16		Lung metastases *n* = 9		Brain metastases *n* = 10		Multiple organ metastases *n* = 10	
% HER2/ECD consistent with imaging conclusion	94		90		100		89		90		100	
% CA 15.3 consistent with imaging conclusion	85		85		94		78		67		90	

Imaging conclusion on metastasis evolution:	progression *n* = 35	regression *n* = 30	progression *n* = 8	regression *n* = 12	progression *n* = 8	regression *n* = 8	progression *n* = 6	regression *n* = 3	progression *n* = 7	regression *n* = 3	progression *n* = 6	regression *n* = 4
% HER2/ECD consistent with imaging conclusion	91	97	88	92	100	100	83	100	86	100	100	100
% CA 15.3 consistent with imaging conclusion	80	90	88	83	88	100	67	100	57	100	100	80

## Discussion

Our study aimed at analyzing a large cohort of patients treated for an invasive breast cancer with overexpression of HER2 status the usefulness of HER2/ECD measurements in the clinical management. The study was monocentric: the inclusion of patients, surgery, follow-up, anatomopathology, and clinical chemistry were done in the same departments during all the studies. Furthermore, we worked with a validated and standardized assay for HER2/ECD measurements as recommended by the FDA. Results showed that HER2/ECD levels at diagnosis were more elevated in advanced breast cancer that HER2/ECD is not a substitute for HER2 status determination, that circulating HER2/ECD levels at diagnosis are significantly correlated with clinical outcome, and that HER2/ECD can be useful in monitoring the efficacy of trastuzumab neoadjuvant treatment and in assisting the early detection of relapse.

At diagnosis, we described a much higher prevalence of elevated HER2/ECD levels in metastatic breast cancers than in non-metastatic ones. In a review, Carney et al. also found that the prevalence of elevated HER2/ECD levels was more frequent in patients with metastatic breast cancer than in women with primary breast cancer [21]. Therefore, the presence of high HER2/ECD levels at diagnosis in patients with HER2 overexpressing breast cancer may be a sign of metastatic disease, and possible distant metastases must be actively searched for. Consequently, early screening for elevated HER2/ECD levels may increase the sensitivity of detecting metastatic cancer.

Some authors have proposed to use serum HER2/ECD concentration as an alternative to histological determination of tissue HER2 status [16, 22]. Our data did not support this idea, as among all tumors of this study with HER2 overexpression, only 15 % of them had an elevated HER2/ECD value (≥15 ng/mL). The lack of correlation between HER2 and HER2/ECD can be explained by the fact that elevated HER2/ECD levels may represent a subgroup of HER2-positive tumors with a higher level of HER2 cleavage and shedding linked to a more aggressive clinical course [23]. The shedding of HER2/ECD that is mediated by matrix metalloproteinases (MMPs) raises potential explanations. Although cancer cells from various tissues can express members of the MMP and ADAM (A Disintegrin and metalloproteinase) families as well as their inhibitors (TIMPs), the major source of these proteinases is from the stroma cells infiltrating the tumor [24]. In our study, elevated levels of HER2/ECD correlated positively with parameters related to tumor aggressiveness, such as vascular invasion, metastatic status, or negativity of estrogen receptors, but not with invaded lymph nodes and progesterone receptor-negative tumors. The literature has frequently reported the correlation between high levels of HER2/ECD and lymph node involvement [25–27]. In our study, lymph nodes were considered as invaded even in cases where there is only a micrometastasis, thus we did not discriminate the invasion intensity and the number of invaded lymph nodes. This probably explains the observed lack of correlation.

We demonstrated that an elevated value of HER2/ECD at diagnosis was strongly associated with a poor overall survival in our cohort of women, and that it was an independent prognostic factor. The link between the HER2/ECD level and survival has several explanations. First, the truncated HER2 form is 10 to 100-fold more oncogenic than the full-length HER2 form [5]. Furthermore, the binding of therapeutic anti-HER2 antibodies on HER2/ECD neutralizes the biological activity of bound anti-HER2 antibodies [28, 29]. Thus, the inhibition of HER2-expressing tumor cells by therapeutic antibodies is limited for patients with high amounts of HER2/ECD. This leads to a more aggressive clinical course for this group of patients. HER2/ECD appears to be a useful biomarker to identify this subgroup of tumors and to improve the risk stratification. This is of great importance as key decisions in the current management of breast cancer involve the determination of prognosis.

This study shows preliminary results indicating that HER2/ECD could also be intended for the monitoring of trastuzumab neoadjuvant treatment efficacy. Patients with a histological complete response showed a significant decrease in the HER2/ECD level that was correlated with the clinical course of disease. This effect was not observed for patients with an incomplete histological response. Few studies have evaluated the variations in levels during a neoadjuvant treatment in patients with tumors overexpressing HER2. Two small prospective studies [30, 31] reported a reduction in HER2/ECD between the neoadjuvant therapy conducted before and 1, 3, and 6 weeks after beginning. This reduction was associated with a complete histological response. Such an impact of trastuzumab therapy on HER2/ECD is explained by the fact that the trastuzumab binding site is near the cleavage site of HER2 [32]. The fixation of trastuzumab to its binding site interferes with HER2 cleavage via steric hindrance of the enzyme substrate interaction [33]. Consequently, trastuzumab induces HER2/ECD decrease by blocking HER2 cleavage. However, trastuzumab could be inefficient due to the absence of trastuzumab fixation on HER2 after elevated HER2/ECD cleavage. In such a situation, a constant or an increasing HER2/ECD level could reveal that the treatment is not effective, and HER2/ECD can be used as an indicator for a second-line therapy based on lapatinib which acts through a different mechanism from that of trastuzumab. Lapatinib induces an inactivation of HER2 tyrosine kinase domain and maximizes trastuzumab-dependent cell cytotoxicity [5]. Another resistance to trastuzumab therapy could be due to the expression of a truncated HER2 form that originally lacks ECD [34]. In such a situation, HER2/ECD would have no interest, and only the immunohistochemical determinations of both HER2/ECD and HER2 intracellular domain (HER2/ICD) could be of interest to observe this truncated form and predict trastuzumab resistance [35].

HER2/ECD can also be appropriate to detect early cancer recurrence as proposed by Carney et al. [33] and as shown in this study, but not in all situations. We observed an isolated increase of HER2/ECD in 18.6 % cases before the diagnosis of relapse or metastasis, and an isolated increase of CA 15.3 in 8.6 % cases before diagnosis of cancer recurrence. Furthermore, HER2/ECD presented a high level of reliability during the monitoring of metastatic breast cancer to predict progression or regression of metastases before medical imaging. This would allow clinicians to make necessary adjustments to drug combination or even prompt them to follow-up patients more frequently. HER2/ECD showed greater concordance than CA 15.3 but the reliability increased when HER2/ECD is concomitantly used with CA 15.3. This result was also observed by Esteva et al. [36]. Consequently, these markers are not interchangeable, but complementary. Indeed, CA 15.3 measures circulating levels of fragments of MUC1/Polymorphic Epithelial Mucin, present on all breast cancer cells, whereas HER2/ECD is a part of HER2.

In this study, the 5 years overall survival was 97.5 % for non-metastatic patients, better than the expected 90 % for such a cohort with HER2 overexpression [37]. Taking into account, the HER2/ECD level allowed an earlier diagnosis of recurrences and consequently more treatment adjustments. Although it is currently difficult to evaluate whether the early treatment by trastuzumab of a HER2 overexpressing non-symptomatic metastasis could increase patient survival, we support the idea that these results stem from using HER2/ECD during the follow-up.

## Conclusion

Our study brings evidences to support the use of HER2/ECD in the routine management of HER2-positive breast cancer patients. At diagnosis, HER2/ECD measurement can be useful to obtain a reference value before treatment, and can be used as a prognosis factor. During the monitoring of neoadjuvant treatment, the evolution of HER2/ECD levels is an informative element to evaluate the therapeutic efficiency. In no case can it be the solely indicator of the treatment efficiency. After clinical remission, HER2/ECD determination is an additional surveillance biomarker helpful for the early diagnosis of relapses. In all cases, the score of a circulating marker measurement should always be interpreted according to the clinical context and the results of other explorations.
